# Intelligent histology for tumor neurosurgery

**DOI:** 10.1093/noajnl/vdag065

**Published:** 2026-02-28

**Authors:** Xinhai Hou, Akhil V Kondepudi, Cheng Jiang, Yiwei Lyu, Edward Samir Harake, Asadur Chowdury, Anna-Katharina Meißner, Volker Neuschmelting, David Reinecke, Gina Fürtjes, Georg Widhalm, Lisa Irinia Körner, Jakob Straehle, Nicolas Neidert, Pierre Scheffler, Jüergen Beck, Michael E Ivan, Ashish H Shah, Aditya S Pandey, Sandra Camelo-Piragua, Dieter Henrik Heiland, Oliver Schnell, Chris Freudiger, Jacob Young, Melike Pekmezci, Katie Scotford, Shawn Hervey-Jumper, Daniel Orringer, Mitchel Berger, Todd Hollon

**Affiliations:** Department of Neurosurgery, University of Michigan, Ann Arbor, Michigan; Department of Neurosurgery, University of Michigan, Ann Arbor, Michigan; Department of Neurosurgery, University of Michigan, Ann Arbor, Michigan; Department of Neurosurgery, University of Michigan, Ann Arbor, Michigan; Department of Neurosurgery, University of Michigan, Ann Arbor, Michigan; Department of Neurosurgery, University of Michigan, Ann Arbor, Michigan; Department of Neurosurgery, University Hospital Cologne, Cologne, Germany; Department of Neurosurgery, University Hospital Cologne, Cologne, Germany; Department of Neurosurgery, University Hospital Cologne, Cologne, Germany; Department of Neurosurgery, University Hospital Cologne, Cologne, Germany; Department of Neurosurgery, Medical University of Vienna, Vienna, Austria; Department of Neurosurgery, Medical University of Vienna, Vienna, Austria; Department of Neurosurgery, University Medical Center Freiburg, Freiburg, Germany; Department of Neurosurgery, University Medical Center Freiburg, Freiburg, Germany; Department of Neurosurgery, University Medical Center Freiburg, Freiburg, Germany; Department of Neurosurgery, University Medical Center Freiburg, Freiburg, Germany; Department of Neurosurgery, University of Miami, Miami, Florida; Department of Neurosurgery, University of Miami, Miami, Florida; Department of Neurosurgery, University of Michigan, Ann Arbor, Michigan; Department of Pathology, University of Michigan, Ann Arbor, Michigan; Department of Neurosurgery, University Hospital Erlangen, Erlangen, Germany; Department of Neurosurgery, University Hospital Erlangen, Erlangen, Germany; Invenio Imaging Inc., Santa Clara, California; Department of Neurosurgery, University of California, San Francisco, California; Department of Neurosurgery, University of California, San Francisco, California; Department of Neurosurgery, University of California, San Francisco, California; Department of Neurosurgery, University of California, San Francisco, California; Department of Neurosurgery, NYU Langone Health, New York, New York; Department of Neurosurgery, University of California, San Francisco, California; Department of Neurosurgery, University of Michigan, Ann Arbor, Michigan

## Abstract

The importance of rapid and accurate histologic analysis of surgical tissue in the operating room has been recognized for over a century. Our standard-of-care intraoperative pathology workflow is based on light microscopy and H&E histology, which is slow, resource-intensive, and lacks real-time digital imaging capabilities. Here, we describe an emerging and innovative method for intraoperative histologic analysis, called Intelligent Histology, that integrates artificial intelligence (AI) with stimulated Raman histology (SRH). SRH is a rapid, label-free, digital imaging method for real-time microscopic tumor tissue analysis. SRH generates high-resolution digital images of surgical specimens within seconds, enabling AI-driven tumor histologic analysis, molecular classification, and tumor infiltration detection. We review the scientific background, clinical translation, and future applications of intelligent histology in tumor neurosurgery. We focus on the major scientific and clinical studies that have demonstrated the transformative potential of intelligent histology across multiple neurosurgical specialties, including neurosurgical oncology, skull base, spine oncology, pediatric tumors, and peripheral nerve tumors. Future directions include the development of AI foundation models through multi-institutional datasets, incorporating clinical and radiologic data for multimodal learning, and predicting patient outcomes. Intelligent histology represents a transformative intraoperative workflow that can reinvent real-time tumor analysis for 21st century neurosurgery.

Key Points

- Intelligent histology uses AI and stimulated Raman histology to provide rapid, accurate, and real-time analysis of surgical tissue, greatly reducing turnaround time compared to traditional frozen section methods.
- AI-powered models not only match but in some cases exceed expert pathologists in diagnosing tumor types, molecular markers, and surgical margins, thus improving decision-making and surgical outcomes.
- The creation of open-source datasets and foundation models encourages multicenter collaboration and facilitates the development of multimodal AI systems, promising broader, more personalized, and efficient pathology workflows in neurosurgery.

The importance of rapid and accurate histologic analysis of surgical tissue in the operating room has been recognized for over a century.^1^ In 1930, Louise Eisenhardt and Harvey Cushing^2^ described a technique for intraoperative histology of tumor specimens. The method has evolved into modern intraoperative pathology, including cytologic preparations and frozen sectioning with hematoxylin and eosin (H&E) staining.^1^ While light microscopy with H&E staining is the standard for intraoperative pathologic analysis, it is labor- and resource-intensive, limited to morphologic diagnoses (eg glial tumors), and does not scale well to serial or multiple specimens. These limitations have hindered histologic analysis from becoming the method of choice for tumor infiltration detection and surgical margin evaluation in neurosurgery. Moreover, because intraoperative H&E histology does not produce real-time digital images, integrating artificial intelligence (AI) for diagnostic decision support has been infeasible.

A major open problem for 21st century neurosurgery is developing innovative methods to improve the intraoperative analysis of surgical tissue. The United States has identified this topic as a major area of innovation for precision healthcare.^3^ Here, we review an emerging method we call “Intelligent Histology,” that combines AI and stimulated Raman histology (SRH), a label-free, nondestructive, optical imaging method, for real-time microscopic specimen analysis, tissue diagnosis, and tumor infiltration detection (Figure 1).^4^ We review the scientific background, clinical translation, and future directions of intelligent histology. Combining state-of-the-art AI and fast optical imaging is an innovative step towards developing a 21st century intraoperative pathology workflow for neurosurgery.

**Figure 1.**
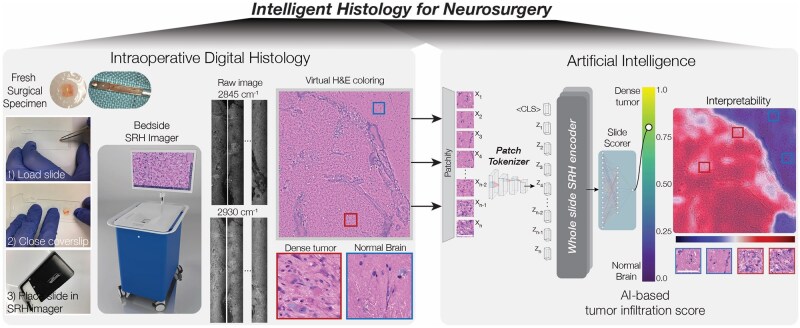
Overview of intelligent histology. (Top left) A fresh, unprocessed neurosurgical specimen is obtained via an open or stereotactic biopsy. (1) The tissue is then loaded into a premade microscope slide, (2) the coverslip is closed, and (3) the slide is placed in the SRH imager (FDA-approved NIO Imaging System, Invenio Imaging, Inc.). (Left center) Raw SRH images are acquired as strips at two Raman shifts, 2845 cm^ − 1^ (lipid channel) and 2930 cm^ − 1^ (protein channel). The time to acquire a 3X3-mm^2^ SRH image is approximately 90 seconds. (Left left) Raw images are colored using a virtual H&E color scheme for clinician review. (Right) AI models can be trained using state-of-the-art deep learning methods for tumor infiltration detection and quantification, for example.^4^ Our intelligent histology models are interpretable because, in addition to providing a whole-slide prediction, region-level attention or prediction offers segmentation of the most diagnostic, tumor-infiltrated regions within an SRH image.

## Background

### Label-Free Imaging with SRH

**Figure 2.**
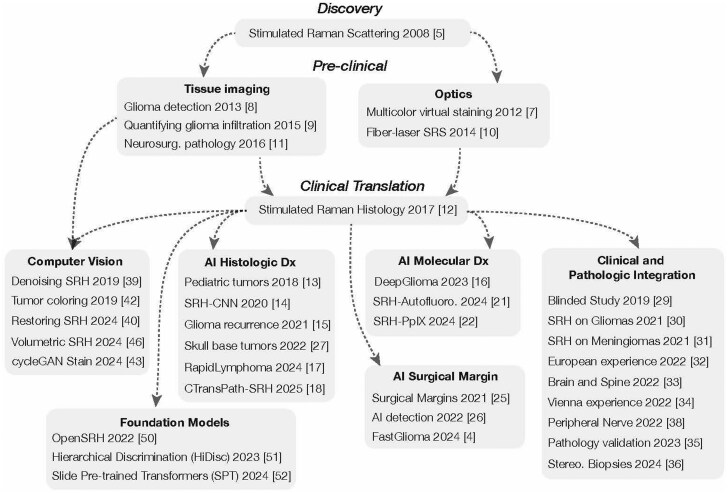
Intelligent histology timeline from (top to bottom) discovery to pre-clinical to clinical translation.

Stimulated Raman scattering is a vibrational microscopy based on Raman scattering that generates high-resolution images without requiring fluorescent labels or dyes. It was first described in 2008 by Freudiger et al. (Figure 2).^5^ A major advantage of stimulated Raman over other optical imaging methods is that the intensity of the Raman signal is directly proportional to the concentration of macromolecules within biological tissue. The differences in biochemical concentrations of lipids, proteins, and nucleic acids within the tissue generate label-free image contrast. While spontaneous Raman spectroscopy has been used in neurosurgery, stimulated Raman has a one-thousand-times greater signal-to-noise ratio for image acquisition and tissue analysis.^6^ The increased signal-to-noise ratio of stimulated Raman has facilitated clinical translation and adoption.

**Figure 3.**
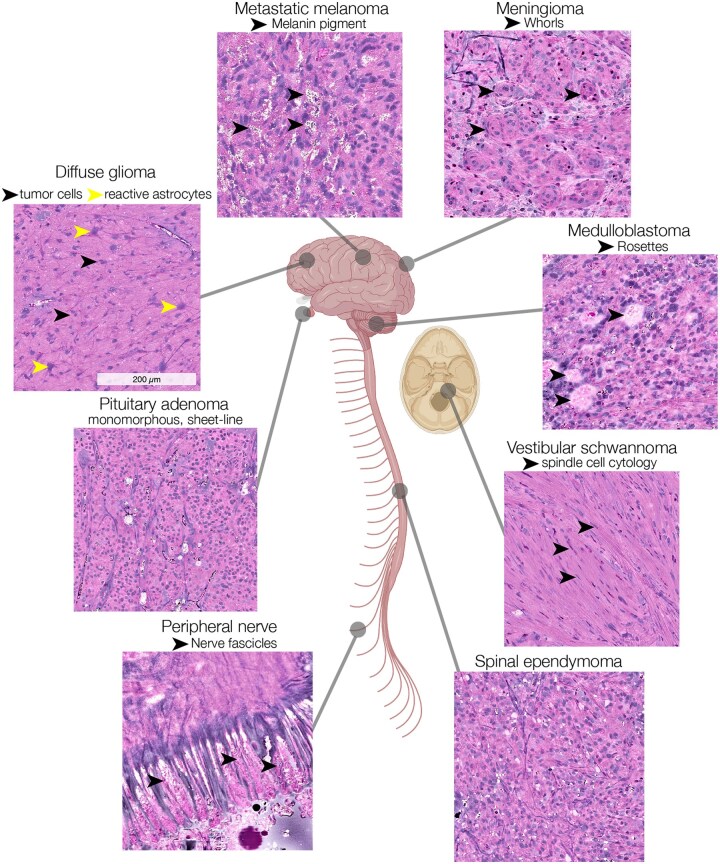
Comprehensive digital histology for tumor neurosurgery. A diverse panel of tumors imaged using rapid, intraoperative SRH. A virtual H&E color scheme is applied for easier intraoperative interpretation for surgeons and pathologists. Pathognomonic features, such as melanin pigment (top left), meningeal whorls (top right), and HomerWright rosettes (right), are seen in SRH images, enabling fast and accurate histologic diagnosis for both clinicians and AI models.

After a sequence of preclinical breakthroughs between 2012 and 2016,^7-11^ Orringer et al^12^ presented the definitive clinical translation of SRH. The clinical SRH imager was a portable fiber-laser-based stimulated Raman scattering microscope used at the bedside to image fresh, unprocessed surgical specimens within minutes of tissue biopsy. SRH captures high-resolution digital images that allow for real-time histologic analysis by pathologists and surgeons. The authors developed a virtual H&E staining method for coloring two-channel, greyscale SRH images that can be used for intraoperative pathology (Figure 3). For intraoperative tumor classification, the pathologists’ diagnoses were concordant when using SRH versus standard H&E pathology. SRH is nonconsumptive and nondestructive: imaged specimens can be sent for additional downstream pathologic analysis, including sequencing and methylation assays, without decreasing diagnostic yield. These results demonstrated the feasibility of SRH workflow for tissue analysis and diagnosis.

### The Beginnings of AI and SRH

SRH is an ideal imaging method for AI because it generates high-resolution, quantitative, and digital images for machine learning. The first application of machine learning to SRH used a linear model to quantify degrees of tumor infiltration based on cellularity, axonal density, and SRH channel intensity ratios.^9^ Subsequent early work used multilayer perceptrons and random forest models on hand engineered SRH features for coarse tumor diagnoses in adult and pediatric brain tumors.^12^^,^^13^ These early applications of machine learning to SRH predate the widespread use of deep learning and the recent explosion of AI foundation models. However, they showed the feasibility of training large-scale SRH-AI models and provided the groundwork for developing intelligent histology.

### Comparison with Other Intraoperative Adjuncts

A range of intraoperative adjuncts have been developed to improve real-time surgical decision-making, but they differ substantially in (1) the type/scale of information provided (macroscopic anatomy vs. microstructure vs. chemical signatures vs. histology-like morphology), (2) workflow burden and time-to-result, and (3) infrastructure and cost. Intraoperative MRI (iMRI) provides whole-brain anatomic and contrast-based assessment of residual disease and can improve extent of resection but requires substantial capital investment and operative workflow integration.^14^ Additionally, the spatial resolution of MRI is not designed to resolve microscopic tumor infiltration. Fluorescence-guided surgery with 5-aminolevulinic acid (5-ALA) is a widely used adjunct for high-grade glioma resection in which administered 5-ALA is metabolized to protoporphyrin IX (PpIX) and emits red fluorescence under blue-violet excitation, providing an intuitive real-time cue to localize tumor and support more complete resection of contrast-enhancing disease.^15^ However, fluorescence intensity can be variable and is often reduced at infiltrative margins or in non-contrast-enhancing or lower-grade components, and it is susceptible to optical attenuation, photobleaching, and confounding by blood or tissue optical properties. Optical micro-imaging approaches such as optical coherence tomography (OCT) and confocal microscopy can provide rapid microstructural information, but they are generally limited by small fields-of-view and constrained imaging depth and/or interpretation burden, and they do not directly provide histology-like context at scale.^16-18^ Chemical approaches, such as Raman spectroscopy^19^^,^^20^ and mass spectrometry,^21^ can offer molecularly informative signatures with fast per-measurement readouts, but are often point-sampled, require specialized equipment, and may not provide the intuitive architectural context of histology. The multitude of intraoperative techniques indicates the importance of rapid tissue evaluation and demonstrates the richness of translational research within this domain. A comparison summary of intraoperative surgical adjuncts can be found in Table 1.

**Table 1.** Comparison of intraoperative diagnostic adjuncts

Modality	Output	Time to feedback	Infrastructure/cost	Main clinical role
Intelligent histology	Digital histology; AI decision support	10-90 seconds	Dedicated SRH system; modest workflow integration	Nondestructive; preserves tissue; closest to pathology-style readout; supports diagnosis, margin, and molecular prediction
Intraoperative MRI (iMRI)	Whole-brain anatomic/contrast imaging	Over 30 minutes, workflow disruption	High capital, specialized suite	Strong for macroscopic residual disease; limited for microscopic infiltration
Fluorescence-guided surgery	Real time fluorescence contrast	Real time	Low incremental (agent + optics)	Highlights candidate tumor regions; sensitivity varies at infiltrative margins; can guide sampling for SRH
Optical micro-imaging (eg conocoal microscopy)	Microstructural imaging	Seconds	Moderate; often investigational	Rapid microstructure; limited FOV/depth and interpretability; does not directly provide histology-like context at scale
Raman spectroscopy	Point-based chemical fingerprints	Seconds per measurement	Probe + spectrometer	Molecularly informative point sampling; lacks histologic architecture; may complement fluorescence/SRH
Mass spectrometry (DESI-MS/REIMS)	Lipid/metabolite profiles	Minutes	Mass spectrometer + specialized operator	High chemical specificity; infrastructure heavy; sampling constraints; typically not a histology-like image readout

Reported performance (accuracy/yield) is indication- and study-dependent; table emphasizes workflow, information type, and feasibility.

Abbreviations: AI, Artificial intelligence; DESI-MS, Desorption electrospray ionization mass spectrometry; FOV, field of vision; REIMS, Rapid evaporative ionization mass spectrometry; SRH, Stimulated Raman histology.

## Clinical Translation of Intelligent Histology

### Histologic Diagnosis of Central Nervous System Tumors

The major role of intraoperative pathology is to analyze surgical tissue and provide a timely histology diagnosis to inform surgical decision-making. A study in 2020 used a convolutional neural network (CNN), trained on over 2.5 million SRH images, to classify the 13 most common histologic diagnoses in neurosurgical oncology.^22^ In a prospective clinical trial, the SRH-CNN achieved a diagnostic accuracy of 93.1%, equivalent to pathologists with standard H&E histology, but an order of magnitude faster (2 vs. 20 minutes). The CNN model learned to identify interpretable histologic features in SRH images, such as hypercellularity, pleomorphism, chromatin structure, and axonal density. Moreover, the SRH-CNN model discovered known histologic features associated with tumor malignancy and progression, such as anaplasia and high nuclei-cytoplasm ratios, and used these to differentiate low grade and high-grade gliomas. A follow-up study fine-tuned the original SRH-CNN model to differentiate tumor recurrence from treatment effect/pseudoprogression.^23^ This task is challenging for intraoperative pathology because previous treatment can induce histologic changes that mimic tumor recurrence, such as gliosis, inflammation, and necrosis. The model achieved a classification accuracy of 95.8% on an external testing cohort of recurrent diffuse gliomas patients.

Differentiating surgical versus non-surgical tumors intraoperatively is essential to define surgical goals. Reinecke et al^24^ developed RapidLymphoma to detect and classify primary central nervous system (CNS) lymphomas using intelligent histology. Because lymphomas are less common than other central nervous system tumors, a major innovation of RapidLymphoma was using self-supervised pretraining, similar to a generative pretrained transformer (GPT). In a prospective, multicenter testing cohort, RapidLymphoma achieved an overall balanced accuracy of 97.8% for detecting primary CNS lymphoma, outperforming frozen sectioning performance. A related innovative work from Scheffle et al used an open-source computational pathology model, CTransPath, for automated SRH feature extraction and lymphoma classification.^25^ To our knowledge, this is the first instance of integrating an off-the-shelf computational pathology model for SRH fine-tuning. Importantly, they achieved comparable classification as RapidLymphoma using a significantly smaller training dataset.

### Molecular Classification of Brain Tumors

**Figure 4.**
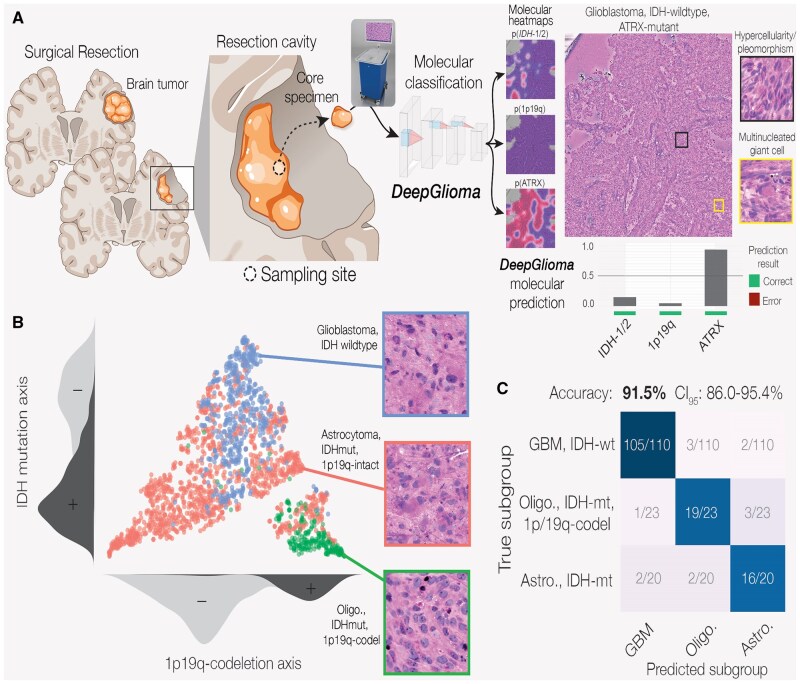
Molecular classification of diffuse gliomas with intelligent histology. (A) A patient with a diffuse glioma undergoes surgical resection. A core specimen is collected and imaged intraoperative using the SRH imager. *DeepGlioma* then performs real-time, automated molecular marker prediction, including isocitrate dehydrogenase-1/2 (IDH) mutation, 1p19q co-deletion, and ATRX mutation, to achieve a final World Health Organization classification within 2 minutes of biopsy. (B) t-Distributed Stochastic Neighbor Embedding (tSNE) plot of *DeepGlioma* outputs shows that the model can differentiate the three major diffuse gliomas subtypes by separating them on an IDH axis and 1p19q axis. (C) *DeepGlioma* classification accuracy surpasses 90% on a prospective, multicenter testing cohort of diffuse glioma patients.^28^

Molecular genetics are now a major factor in classifying CNS tumors.^26^ Adult-type diffuse gliomas are classified by isocitrate dehydrogenase-1/2 (IDH) mutations and 1p19q co79 deletions (1p19q). Importantly, Hervey-Jumper et al^27^ demonstrated that surgical goals, such as gross total versus supratotal resection, should be informed by IDH and 1p19q status. Unfortunately, conventional intraoperative pathology does not provide molecular data. Intelligent histology has the potential to predict molecular markers from SRH images to inform surgical goals. DeepGlioma was developed in 2023 for rapid and accurate molecular classification of diffuse gliomas (Figure 4).^28^ DeepGlioma was the first multimodal intelligent histology model trained with SRH and large-scale, public genomic data. It was also first trained to learn the genomic landscape of diffuse gliomas, such as co-occurrence statistics of IDH mutations and 1p19q co-deletions. Then, the model was trained to use this landscape to classify gliomas based on SRH image features. In a prospective, multicenter, international testing cohort of patients with diffuse glioma (*N* = 153), DeepGlioma achieved a mean accuracy of 93.3 ± 1.6% for predicting the molecular markers used by the World Health Organization to define diffuse gliomas, including IDH mutation, 1p19q co-deletion, and ATRX mutation.

Intelligent histology has characterized two-photon fluorescence in surgical specimens.^29^^,^^30^ The SRH-CNN model^22^ was used to identify tumor infiltrated regions in a diverse set of surgical specimens. Spatially registered two-photon fluorescence images were acquired and the mean autofluorescence was measured within AI-selected tumor infiltrated regions. The authors found autofluorescence in the brain varies depending on the tissue type and localization and differs significantly among various brain tumors. A subsequent study elucidated the molecular and spatial relationship between SRH image features and protoporphyrin IX (PpIX) fluorescence due to 5-aminolevulinic acid (5-ALA) used in glioma surgery.^30^ Using 115 high-grade glioma patients from four medical centers, the authors discovered 5 distinct patterns of PpIX fluorescence using semi-supervised learning. Spatial transcriptomic analyses of the imaged tissue demonstrated that myeloid cells predominate in areas where PpIX accumulates in the intracellular space. Additional analysis of spatially resolved metabolomics, transcriptomics and RNA-sequencing data confirmed that myeloid cells preferentially accumulate and metabolize PpIX. These findings demonstrate how intelligent histology can be used to shed new light on existing surgical tools and the immune microenvironment of gliomas.

### Surgical Margin Analysis

Safe maximal resection has stood the test of time as an essential component in the management of brain tumors.^27^ Residual tumor burden after surgery is the major risk factor for a worse overall prognosis in the majority of brain tumor diagnoses. Unfortunately, clinical studies have demonstrated that many patients, perhaps the majority, do not receive optimal surgical treatment.^31^ Dense, safely resectable tumor infiltration is found at the surgical margin in 30% of diffuse gliomas patients.^32^ Developing on previous work, Reinecke et al^33^ showed that histologic classification models can be leveraged for tumor infiltration detection and validated this approach in a large external cohort.

**Figure 5.**
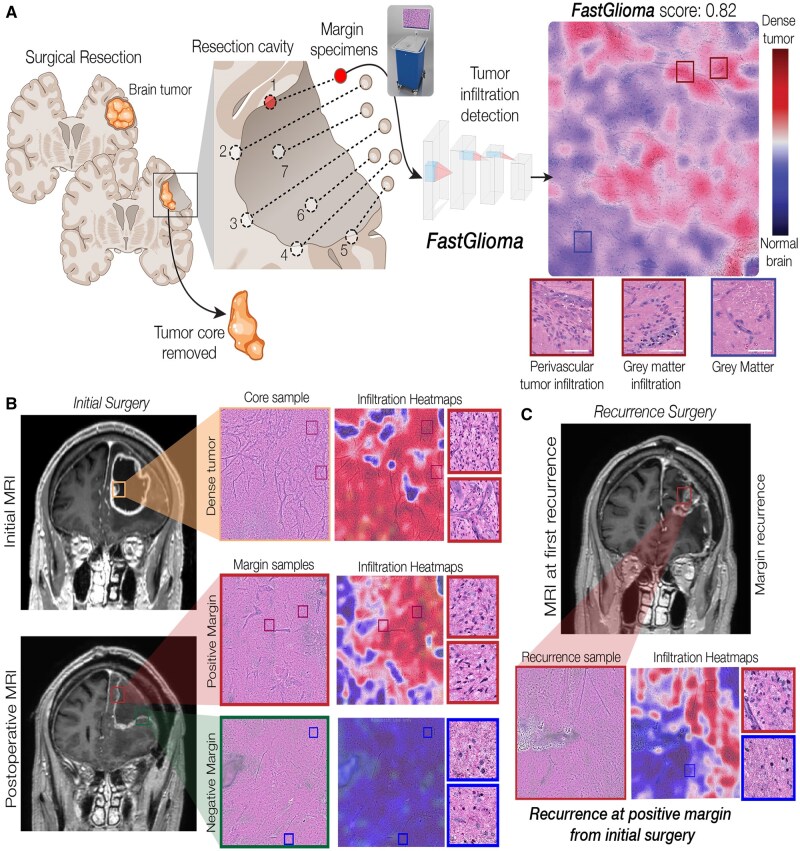
Tumor infiltration detection with intelligent histology. (A) A patient with a brain tumor undergoes surgical resection. Following resection of the tumor core, margin specimens are collected for SRH imaging and AI-based detection of tumor infiltration. *FastGlioma* outputs a heatmap that identifies regions of tumor infiltration and a normalized score between 0 (normal) and 1 (dense tumor). (B) An illustrative case of a patient who underwent *FastGlioma*-guided tumor resection at their initial surgery. A positive margin was identified at the medial border of the resection cavity. (C) Patient subsequently presented with a recurrence along the medial resection cavity at the positive margin site. These results demonstrate that *FastGlioma* can detect microscopic tumor burden and guide surgical resections, potentially prolonging progression-free and overall survival.

FastGlioma is an intelligent histology model for fast (<10 seconds) and accurate detection of glioma infiltration at the surgical margin (Figure 5).^4^ FastGlioma was pretrained on a large-scale SRH dataset, around 4 million images, using self-supervision (ie without labels). The model was then fined-tuned on a small, expert-annotated tumor infiltration dataset to output a tumor infiltration score between 0 and 1, where 0 was normal brain and 1 was dense tumor.^32^ In a prospective, multicenter, international testing cohort of patients with diffuse glioma (*N* = 220), FastGlioma was able to detect and quantify the degree of tumor infiltration with an average area under the receiver operating characteristic curve of 92.1 ± 0.9%. FastGlioma outperformed image-guided and fluorescence-guided adjuncts for detecting tumor infiltration during surgery by a wide margin in a head-to-head, prospective study (*N* = 129). Only 3.8% (5 out of 129) of patients in the FastGlioma arm had dense residual tumor compared with 24.0% (31 out of 129) in the surgical adjuncts arm. We encountered no adverse events with surgical margin sampling. FastGlioma was performant across all WHO diffuse glioma molecular subtypes. Additionally, FastGlioma showed generalization to other adult and paediatric brain tumor diagnoses, such as metastatic tumors and diffuse midline gliomas, demonstrating the potential of FastGlioma as a general-purpose adjunct for guiding brain tumor surgeries.

The clinical importance of surgical margin analysis is not limited to diffuse gliomas. Residual tumor burden after meningioma or pituitary surgery, for example, is known to be a strong predictor of future recurrence. Several groups have shown the feasibility of using Raman spectroscopy to detect meningioma and metastatic tumor infiltrations in surgical specimens.^34^^,^^35^ Jiang et al^36^ showed that microscopic tumor infiltration can be detected using intelligent histology in grossly normal dura at the resection margin of meningiomas. In pituitary adenoma surgery, the authors showed that submillimeter adrenocorticotropic hormone (ACTH)-secreting pituitary adenomas can be detected in intraoperative pituitary specimens. Furthermore, skull base surgeons have highlighted the potential of intelligent histology in sampling the medial wall of the cavernous sinus for adenoma invasion during pituitary surgery to offer real-time feedback on surgical decision-making.^37^

### Integrating Intelligent Histology into Neurosurgery

A global effort has emerged towards integrating SRH and intelligent histology into neurosurgical practice. Multiple independent groups have shown the benefits of using SRH in the operating room to rapidly evaluate surgical specimens. In a prospective blinded study, researchers at the University of Miami demonstrated that SRH-based diagnosis was on average 30 minutes faster than frozen section-based diagnoses, with near perfect diagnostic concordance (Cohen’s k = 0.834, *P *< .0001) between SRH and permanent/frozen sections.^38^ They subsequently published their clinical SRH experience in meningiomas and gliomas.^39^^,^^40^ Paired publications from researchers at the University of Freiburg reported their experience integrating SRH into a large European neurosurgical center, highlighting comparable diagnostic accuracy in SRH/H&E concordance with both spine and brain tumors.^41^^,^^42^ Researchers at the University of Vienna presented their experience with intraoperative SRH across a wide range of brain tumors and non-neoplastic lesions, such as cavernomas and epidermoid cysts. Importantly, they showed excellent concordance between SRH and H&E to assess degrees of cellularity and tumor infiltration at the surgical margin.^43^

Movahed-Ezazia et al^44^ performed a 1-year study at New York University to evaluate the median turnaround time (TAT) of conventional neuropathology frozen sections with prospective SRH. The median TAT for SRH diagnosis was 3.7 minutes, approximately 10× faster than the median frozen section TAT, 31 minutes. Their findings support the feasibility and time-savings of implementing SRH as a rapid method for intraoperative neuropathology. Reinecke et al presented a comprehensive study from the University of Cologne on using intelligent histology for stereotactic brain biopsies, incorporating both histologic and molecular classification models.^22^^,^^28^^,^^45^ Firstly, they demonstrated that intelligent histology yields accurate predictions on stereotactic brain biopsies. Critically, they showed that biopsy sample size effects diagnostic accuracy and systematically found a minimal size/surface area required for accurate diagnosis. This was the first study to evaluate the relationship between tissue specimen size and model performance. Meißner et al^46^ then showed that frozen tissue samples from a tissue biobank can be used to generate high-quality images for AI training, thereby increasing data examples of rare tumors and diagnoses.

Finally, intelligent histology has been applied to peripheral nerve tumor surgery.^47^ SRH effectively visualizes lipids and may be well-suited for intraoperative assessment of peripheral nerve quality. Wilson et al.^47^ used SRH intraoperatively on 6 peripheral nerve cases to generate high-resolution images of myelinated axons within nerve fascicles. Their results suggest that SRH provides a reliable and quantitative method for evaluating myelin in peripheral nerves, potentially improving surgical decision-making for nerve repair.

### Biomedical Computer Vision

Intelligent histology has driven new developments in AI-based biomedical microscopy and computer vision. A major area has been improving SRH image quality through denoising, restoration, colorization, and super-resolution. Manifold et al.^48^ trained a U-Net model to denoise SRH images acquired at low laser power. Subsequent work developed a generative SRH image denoising and restoration method using diffusion models called restorative step-calibrated diffusion (RSCD).^49^ RSCD can take any SRH image, with unknown sources or severity of image degradation, and restore the image to diagnostic quality for pathologic review and AI prediction. With advances in image-to-image models,^50^ virtual coloring of SRH images has improved. Shin et al^51^ showed that tailored coloring, beyond virtual H&E staining, that utilizes rich SRH chemical information can improve diagnostic accuracy of skull base tumors. In 2024, Liu at al^52^ trained a coloring generative adversarial network to convert greyscale SRH images to an H&E coloring perceptually indistinguishable from laboratory H&E staining. The role of 3D microscopy in biomedical research and computational pathology is growing.^53^^,^^54^ Jiang et al^55^ presented the first intelligence histology model to generate high-resolution 3D SRH images. Using a generative diffusion model, 2D SRH images are converted into 3D images that better capture cellular morphology, vascularity, and tumor infiltration. A summary of all key intelligent histology publications is shown in Table 2.

**Table 2.** Summary of key publications in intelligent histology

Authors & Year	Title	Major contribution
Freudiger et al, 2008^5^	Label-free biomedical imaging with high sensitivity by stimulated Raman scattering microscopy	First description and demonstration of SRS microscopy for high-sensitivity, label-free biomedical imaging-foundational optics enabling later SRH workflows
Ji et al, 2013^8^	Rapid, label-free detection of brain tumors with stimulated Raman scattering microscopy	Showed that SRS microscopy can rapidly detect brain tumors in a label-free manner, establishing feasibility for neurosurgical tumor detection
Freudiger et al, 2014^10^	Stimulated Raman scattering microscopy with a robust fibre laser source	Demonstrated a robust fiber-laser source for SRS microscopy, advancing practicality and clinical translation of SRS/SRH imaging systems
Ji et al, 2015^9^	Detection of human brain tumor infiltration with quantitative stimulated Raman scattering microscopy	Quantitatively detected tumor infiltration in human brain tissue using SRS, moving beyond tumor detection to infiltration assessment
Orringer et al, 2017^12^	Rapid intraoperative histology of unprocessed surgical specimens via fibre-laser-based stimulated Raman scattering microscopy	Established SRH as rapid intraoperative histology for unprocessed specimens using fiber-laser SRS, key step enabling routine intraoperative SRH
Hollon et al, 2018^13^	Rapid intraoperative diagnosis of pediatric brain tumors using stimulated Raman histology	Applied SRH to rapid intraoperative diagnosis in pediatric brain tumors, extending SRH clinical impact to pediatric neurosurgery
Eichberg et al, 2019^38^	Stimulated Raman histology for rapid and accurate intraoperative diagnosis of CNS tumors: prospective blinded study	Prospective blinded clinical study demonstrating rapid and accurate intraoperative SRH diagnosis of CNS tumors in a real-world workflow
Shin et al, 2019^51^	Intraoperative assessment of skull base tumors using stimulated Raman scattering microscopy	Demonstrated intraoperative SRS/SRH assessment of skull base tumors, expanding SRH beyond glioma and supporting broader neurosurgical pathology applications
Hollon et al, 2020^22^	***SRH-CNN:*** Near real-time intraoperative brain tumor diagnosis using stimulated Raman histology and deep neural networks	Seminal integration of SRH with deep neural networks for near-real-time intraoperative brain tumor diagnosis-establishing AI histologic diagnosis from SRH at scale
Pekmezci et al, 2021	Detection of glioma infiltration at the tumor margin using quantitative stimulated Raman scattering histology	Demonstrated quantitative SRH/SRS methods for detecting glioma infiltration at surgical margins, directly addressing margin assessment
Neidert et al, 2022^41^	Stimulated Raman histology in the neurosurgical workflow of a major European neurosurgical center—part a	Large European center integration of SRH into neurosurgical workflow (part A), supporting feasibility and operationalization in routine practice
Reinecke et al, 2022^33^	Novel rapid intraoperative qualitative tumor detection by a residual convolutional neural network using label-free stimulated Raman scattering microscopy	Introduced a residual CNN approach for rapid qualitative tumor detection on label-free SRS/SRH images, contributing to AI-assisted intraoperative detection
Jiang et al, 2022^36^	Rapid automated analysis of skull base tumor specimens using intraoperative optical imaging and artificial intelligence	Extended AI + intraoperative optical imaging (SRH context in the timeline) to skull base tumor specimens for rapid automated analysis
Jiang et al, 2022^56^	OpenSRH: optimizing brain tumor surgery using intraoperative stimulated Raman histology	Released OpenSRH, a public clinical SRH dataset enabling benchmarking, reproducibility, and foundation-model style pretraining for SRH
Wadiura et al, 2022^43^	Toward digital histopathological assessment in surgery for central nervous system tumors using stimulated Raman histology	Reported Vienna experience moving toward digital intraoperative histopathologic assessment using SRH across CNS tumors and non-neoplastic lesions
Hollon et al, 2023^28^	***DeepGlioma*** *:* Artificial-intelligence-based molecular classification of diffuse gliomas using rapid, label-free optical imaging	Demonstrated AI-based molecular classification of diffuse gliomas from rapid, label-free optical imaging (SRH), linking SRH to molecular diagnostics
Jiang et al, 2023^57^	Hierarchical discriminative learning improves visual representations of biomedical microscopy	Introduced HiDisc: A self-supervised hierarchical discriminative learning strategy (patch/slide/patient) for stronger SRH representations and foundation model training
Nasir-Moin et al, 2024^30^	Localization of protoporphyrin IX during glioma-resection surgery via paired stimulated Raman histology and fluorescence microscopy	Demonstrated paired SRH and fluorescence microscopy for intraoperative PpIX localization during glioma surgery, linking SRH to fluorescence-guided resection biology
Reinecke et al, 2024^24^	***RapidLymphoma***: Fast intraoperative detection of primary CNS lymphoma and differentiation from common CNS tumors using stimulated Raman histology and deep learning	Applied SRH + deep learning to rapidly detect primary CNS lymphoma and differentiate it from other CNS tumors, expanding AI-SRH diagnostic scope
Reinecke et al, 2024^45^	Streamlined intraoperative brain tumor classification and molecular subtyping in stereotactic biopsies using stimulated Raman histology and deep learning	Demonstrated SRH + deep learning for stereotactic biopsies including histologic classification and molecular subtyping, highlighting sample-size effects and biopsy workflow relevance
Kondepudi et al, 2025^4^	***FastGlioma***: Foundation models for fast, label-free detection of glioma infiltration	Demonstrated foundation-model pretraining for SRH enabling fast, label-free glioma infiltration detection-showing benefits of large-scale pretraining then task fine-tuning

Bold, italicized text indicates models mentioned by name in the manuscript.

Abbreviations: AI, artificial intelligence; CNN, convolutional neural network; CNS, central nervous system; HiDisc, Hierarchical discriminative; PpIX, protoporphyrin IX; SRH, stimulated Raman histology; SRS, stimulated Raman scattering.

### Current Limitations

Current SRH systems balance speed and image quality, but image acquisition remains a practical bottleneck when many specimens or large margins must be evaluated. Continued progress will require faster acquisition through improved scanning hardware and parallelization while preserving diagnostic fidelity. A second limitation is multiplexing: most clinical SRH implementations emphasize two-channel contrast to support virtual H&E-like visualization, but richer chemical contrast could improve interpretability and enable new intraoperative readouts.^58^ Achieving this will likely require more efficient spectroscopic or multiplexed strategies that do not meaningfully slow imaging. Finally, broader clinical adoption will benefit from multispecimen imaging platforms that allow multiple specimens to be loaded and imaged within a single slide or cartridge, reducing repeated handling and accelerating throughput in high-volume operative workflows.

On the AI side, most current intelligent histology models remain specialized and unimodal, trained for a single task using SRH images alone; for example, diagnosis, infiltration scoring, or molecular prediction, which motivates the next-generation shift toward foundation models that can be adapted across tasks and institutions.^59^ A related challenge is algorithmic bias and distribution shift, where performance can vary with differences in patient populations, tumor prevalence, sampling patterns, slide preparation, or imaging hardware.^60^^,^^61^ While recent multicenter prospective validation has demonstrated strong performance and even favorable head-to-head comparisons versus existing intraoperative adjuncts in specific settings, robust deployment will require systematic auditing for failure modes, calibration drift, and subgroup performance disparities, with transparent reporting and continual external validation as datasets expand.

## Future Directions of Intelligent Histology

### Foundational Models for Intelligent Histology

Foundations models are any AI model trained in broad, diverse, and minimally unorganized data, generally using self-supervision (ie no labels), that can be adapted or fine-tuned to a wide range of downstream tasks. Examples of foundational models in other domains include large language models such as the GPT series finetuned conversation to develop ChatGPT,^62^ and vision models such as CLIP finetuned for text-to-image generation to develop DALL-E.^63^^,^^64^ Foundation modeling is the leading candidate for developing generalist medical AI.^59^ A major future direction of intelligent histology is developing foundation models to solve multiple diagnostic or prognostic tasks. Previous work has trained models for a single task on a curated dataset, such as histologic diagnosis or tumor infiltration quantification. Foundation model research has shown that training models with larger datasets, followed by fine-tuning, results in better generalization and performance.^65^^,^^66^ These results motivated the large-scale pre-training of FastGlioma before tumor infiltration fine-tuning.^4^

To promote open science and the development of foundation models for intelligent histology, we released two open-source resources: OpenSRH and ELUCIDATE. OpenSRH is the first public dataset of clinical SRH images from 300+ brain tumors patients and 1300+ unique whole slide SRH images.^56^ OpenSRH contains data from the most common brain tumors diagnoses, full pathologic annotations, whole slide tumor segmentations, and both raw and processed images. We hope to foster multi-institutional datasets that will serve as the basis for training foundation models. Incorporating OpenSRH, Jiang, Hou, et al. published two strategies for foundation model training that address SRH region-level training, called HiDisc^57^ and whole slide-level training, called Slide Pre-trained Transformers (SPT) (see Supplementary Figure S1).^67^ Both HiDisc and SPT scale to large datasets and use self-supervision only to train intelligent histology models. ELUCIDATE is a web-based protocol to annotate and automate single-cell instance segmentation for SRH images.^68^ Future directions include expanding foundational models to include single-cell computer vision tasks. We aim to develop multi-institutional consortia that will facilitate SRH data sharing and foundation model development.

### Multimodal Learning

Multimodal models can accept input data from two or more domains. Multimodal models, such as DALL-E^69^ and LLaVa,^70^ can take images or text as input and output images or text. The major advantage of multimodal learning in medical AI is that most diagnostic or prognostic tasks should include text, tabular, and image data to improve clinical context. Pathologists and radiologists use clinical context when providing pathology or radiology reports. Recent computational pathology models have embraced multimodal learning to improve performance across multiple cancer types and include visual question answering.^71^ For example, interpreting SRH images of brain tumors will be aided by knowing the patient’s age, presenting symptoms, tumor location, and MRI features. Including these additional variables for clinical context will improve overall performance and prevent diagnostic errors. Future intelligent histology models will benefit from being multimodal to better replicate how clinical decisions incorporate information from multiple sources.

### Patient Outcome Prediction

Predicting patient outcomes is arguably the most important AI task in precision medicine. Prognostication, or forecasting future events from past and present data, is one of the more challenging machine learning tasks due to limited data and unforeseen or random events. Recent computation pathology research has studied stratifying patient cancer survival from H&E images and genetic data.^72^^,^^73^ While this work represents an important step forward, this strategy will always produce suboptimal results due to not accounting for important prognostic information, such as preoperative tumor volumes, extent of resection, functional status, age, etc. This reemphasizes the essential role of multimodal models for patient outcome prediction. Current multimodal intelligent histology research is underway to incorporate patient demographic, clinical, radiologic, surgical, and pathologic information to stratify survival for diffuse glioma patients.

## Conclusions

Intelligent histology unifies rapid digital histology and deep learning to advance intraoperative neuropathology. Neurosurgeons now have access to timely and reliable AI-based interpretation of tumor type, molecular profile, and margin status at the patient’s bedside, enabling safer, more complete resections and informed surgical decision-making. Successive intelligent histology breakthroughs chart a clear trajectory toward more comprehensive and interpretable support systems that generalize across diverse tumor types and operative workflows. Open-source resources like OpenSRH, together with early self-supervised foundation models, signal a collaborative shift toward large-scale, multimodal, multicenter AI development and validation. Collectively, these advances demonstrate a transformative intraoperative pathology paradigm in which every fresh specimen becomes a rich digital substrate for AI, accelerating diagnosis, guiding precision surgery, and deepening our understanding of tumor biology for the benefit of patients and our neurosurgical community.

## Supplementary Material

vdag065_Supplementary_Data

## Data Availability

Data are available within the article and its supplementary materials.
