# Supplementary material for: Intelligent histology for tumor neurosurgery
Source: Neurooncol Adv. 2026 Feb 28;8(1):vdag065. doi: 10.1093/noajnl/vdag065 (PMC13047285; doi:10.1093/noajnl/vdag065)
Supplement: vdag065_Supplementary_Data [file vdag065_supplementary_data.zip › Supplementary_Figure_Legend.docx]

Supplementary Material

**Supplementary Fig. S1.** Foundation models for intelligent histology. **(**A) Clinical biomedical microscopy and SRH has a hierarchical patch-slide-patient data structure. This structured can be used to train foundation models without the need to use data labels. Hierarchical discrimination (HiDisc) combines patch, slide, and patient discrimination into a unified self-supervised learning task that can be used to train intelligent histology foundation models. (B) After training with HiDisc, we show the t-Distributed Stochastic Neighbor Embedding (tSNE) plot of stimulated Raman histology (SRH) images with the diagnosis labels. Note that the model was not trained with labels yet learns the differentiate the major tumor types in neurosurgery. Moreover, it captures diversity and heterogeneity for each specimen (right). The model captures unique features for each patient, demonstrated by the clustering of regions from the same patient.
