# Supplementary figures and images for: Intelligent histology for tumor neurosurgery
Source: Neurooncol Adv. 2026 Feb 28;8(1):vdag065. doi: 10.1093/noajnl/vdag065 (PMC13047285; doi:10.1093/noajnl/vdag065)

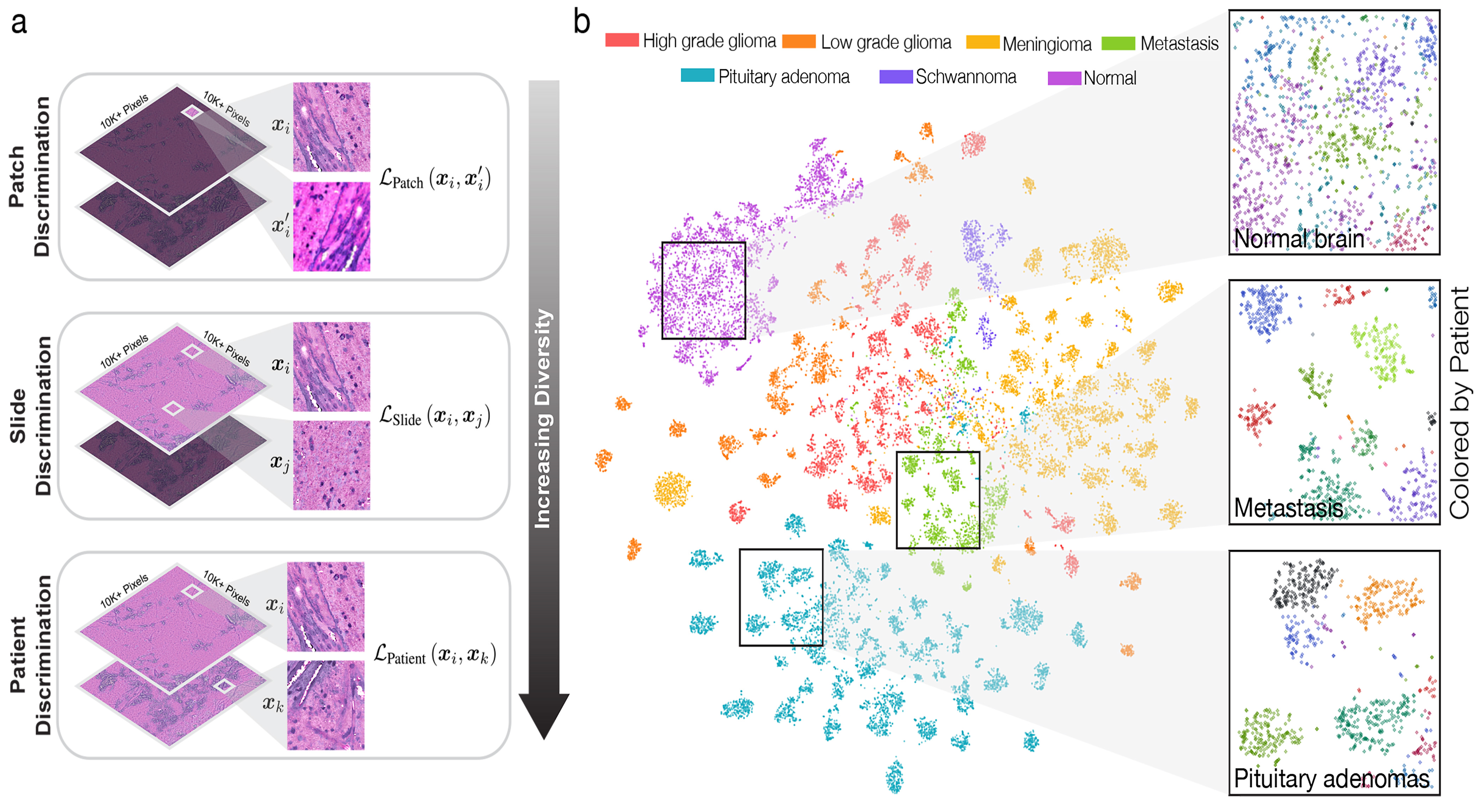

Supplement: vdag065_Supplementary_Data [file vdag065_supplementary_data.zip › Supplementary_Figure_1.tif]
